# Simultaneous targeting of Tim3 and A2a receptors modulates MSLN-CAR T cell antitumor function in a human cervical tumor xenograft model

**DOI:** 10.3389/fimmu.2024.1362904

**Published:** 2024-05-24

**Authors:** Tahereh Soltantoyeh, Behnia Akbari, Zahra Shahosseini, Hamid Reza Mirzaei, Jamshid Hadjati

**Affiliations:** ^1^ Department of Medical Immunology, School of Medicine, Tehran University of Medical Sciences, Tehran, Iran; ^2^ Department of Medical Biotechnology, School of Allied Medical Sciences, Iran University of Medical Sciences, Tehran, Iran; ^3^ Virology Department, Pasteur Institute of Iran, Tehran, Iran

**Keywords:** CAR T cell therapy, solid tumors, genetic targeting, xenograft, A2aR, Tim3, shRNA

## Abstract

**Introduction:**

Chimeric antigen receptor (CAR) T cell therapy has transformed the treatment of hematological malignancies. However, its efficacy in solid tumors is limited by the immunosuppressive tumor microenvironment that compromises CAR T cell antitumor function in clinical settings. To overcome this challenge, researchers have investigated the potential of inhibiting specific immune checkpoint receptors, including A2aR (Adenosine A2 Receptor) and Tim3 (T cell immunoglobulin and mucin domain-containing protein 3), to enhance CAR T cell function. In this study, we evaluated the impact of genetic targeting of Tim3 and A2a receptors on the antitumor function of human mesothelin-specific CAR T cells (MSLN-CAR) *in vitro* and *in vivo.*

**Methods:**

Second-generation anti-mesothelin CAR T cells were produced using standard cellular and molecular techniques. A2aR-knockdown and/or Tim3- knockdown anti-mesothelin-CAR T cells were generated using shRNA-mediated gene silencing. The antitumor function of CAR T cells was evaluated by measuring cytokine production, proliferation, and cytotoxicity *in vitro* through coculture with cervical cancer cells (HeLa cell line). To evaluate *in vivo* antitumor efficacy of manufactured CAR T cells, tumor growth and mouse survival were monitored in a human cervical cancer xenograft model.

**Results:**

*In vitro* experiments demonstrated that knockdown of A2aR alone or in combination with Tim3 significantly improved CAR T cell proliferation, cytokine production, and cytotoxicity in presence of tumor cells in an antigen-specific manner. Furthermore, in the humanized xenograft model, both double knockdown CAR T cells and control CAR T cells could effectively control tumor growth. However, single knockdown CAR T cells were associated with reduced survival in mice

**Conclusion:**

These findings highlight the potential of concomitant genetic targeting of Tim3 and A2a receptors to augment the efficacy of CAR T cell therapy in solid tumors. Nevertheless, caution should be exercised in light of our observation of decreased survival in mice treated with single knockdown MSLN-CAR T cells, emphasizing the need for careful efficacy considerations.

## Background

T cells can be engineered to express chimeric antigen receptors containing single-chain variable fragments (scFv) specific to tumor antigens as extracellular regions, transmembrane domains and appropriate intracellular signaling domains (1). Although the use of CAR T cells as a type of adoptive T cell therapy has achieved unprecedented success in hematologic malignancy, however, its success in solid tumors has been marginal more likely due to the presence of a hostile tumor microenvironment (TME). Several factors, including immunosuppressive cells and molecules (such as Tregs; regulatory T cells, MDSCs; Myeloid-derived suppressor cells, and TGF-β; Transforming growth factor-β), nutrient deprivation and hypoxia in the TME, induce the expression of inhibitory receptors such as Tim3, PD-1 (Programmed cell death protein 1), LAG3 (Lymphocyte-activation gene 3), TIGIT (T cell immunoreceptor with Ig and ITIM domains) and A2aR on the surface of T cells where they home into such a harsh microenvironment (2, 3). Hypoxia in the TME activates the HIF-1α (Hypoxia inducible factor-1α) pathway, which upregulates the expression of the ectonucleotidases CD39 and CD73 on tumor cells and immune cells. The ectonucleotidases sequentially lead to increased extracellular adenosine (4). Among four different receptors for adenosine, including A1, A2a, A2b, and A3 receptors, the A2a receptor on T cells binds to adenosine with a higher affinity and has inhibitory roles (5). Upon binding of adenosine to the A2a receptor on T cells, the G protein coupled to the A2a receptor mediates intracellular signaling through increased cAMP, which thereby inhibits TCR signaling and attenuates the antitumor function of T cells, including cytokine production, cytotoxicity and proliferation (6). Tim3 expression, as a part of the inhibitory checkpoint module, is increased on exhausted tumor-infiltrating T cells (7, 8). Tim3 ligand engagement through uncoupling of HLA-B-associated transcript 3 (Bat3) and recruitment of CD45 and CD148 phosphatase prevents phosphorylation of Tim3 tyrosine motifs Y256 and Y263 and LCK and eventually leads to inhibition of TCR signaling (9). Previous studies by Jafarzadeh et al. and Masoumi et al. have shown that targeting the Tim3 or A2a receptor can improve the *in vitro* antitumor function of mesothelin-specific CAR T cells (MSLN-CAR T cells) (10, 11). Since each inhibitory receptor reduces the antitumor functions of T cells through distinct signaling pathways (12), in this study, we aimed to generate Tim3/A2aR double knockdown MSLN-CAR T cells using lentiviral cotransduction. We then performed *in vitro* functional assays to evaluate the proliferation, cytotoxicity, and cytokine production of MSLN-CAR T cells, Tim3 or A2aR single knockdown (KD), and Tim3/A2aR double knockdown MSLN-CAR T cells. Additionally, we investigated *in vivo* antitumor effects of different MSLN-CAR T cells (MSLN-CAR T cells, Tim3.KD. MSLN-CAR T cells, A2aR.KD. MSLN-CAR T cells and Tim3/A2R.KD MSLN-CAR T cells) in tumor-bearing C57BL/6-nude mice.

## Methods

### Cell lines and culture

The HeLa, Panc-1, HEK293T, and Jurkat cell lines were obtained from the Iranian Biological Resource Center (IBRC) and confirmed to be free of mycoplasma contamination. The HeLa cell line was used as a mesothelin-positive cell line, while the Panc-1 was used as a mesothelin-negative cell line. The expression of mesothelin was verified by flow cytometry using a PE-conjugated anti-human mesothelin antibody (R&D Systems, Minneapolis, MN, USA). The HEK293T cell line was used as a packaging cell line for the production of lentiviral particles. Prior to generating CAR T cells, the Jurkat cell line was used for the titration of lentivirus. HEK293T, HeLa, and Panc-1 cells were cultured in DMEM (Gibco, Life Technologies) with 10% fetal bovine serum (FBS, Gibco, Life Technologies), 1% penicillin/streptomycin, and 2 mmol L-glutamine (Gibco, Life Technologies) and incubated at 37°C in 5% CO2. The Jurkat cell line was cultured in RPMI1640 (Gibco, Life Technologies) with 25 mmol/L HEPES (Sigma Aldrich), 10% FBS, 1% penicillin/streptomycin, and 2 mmol L-glutamine and incubated at 37°C in 5% CO2.

### Lentiviral vector production, concentration and titration

**Figure 1:**
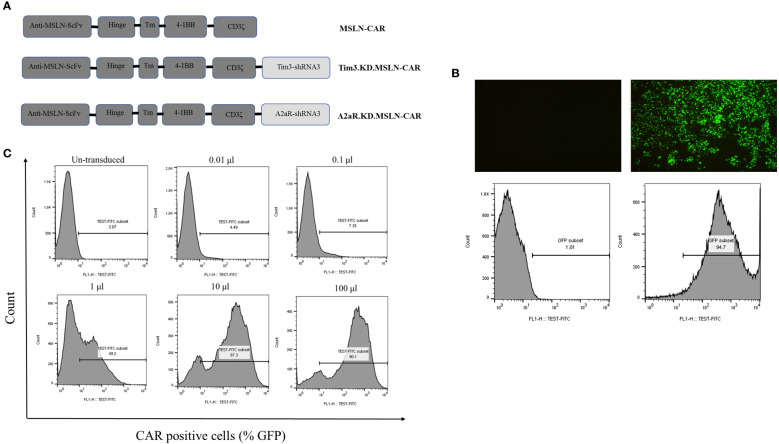
Lentiviral vector production and titration. Schematic representation of three second-generation CAR constructs **(A)**. MSLN-CAR contains an anti-mesothelin (MSLN) scFv, human CD8α-derived hinge, 4–1BB-derived transmembrane (TM) domain, and two intracellular domains, including 4–1BB and CD3ζ. A2aR.KD and Tim3.KD.MSLN-CAR constructs include an anti-A2aR or Tim3 shRNA sequence after the CD3ζ sequence. ShRNA sequences were designed to target the A2aR and Tim3 genes. Fluorescence microscopy images and histogram plots show the percentage of HEK293T cells transfected with lentiviral vectors containing GFP (right) compared to untransfected HEK293T cells (left) **(B)**. Flow cytometry histograms show the titration of concentrated virus-containing media (VCM) on 1×10^6^ Jurkat cells/well **(C)**. The percentage of CAR-expressing Jurkat cells (GFP%) three days after transduction with 0.01, 0.1, 1, 10, and 100 μL of VCM is shown.


To generate second-generation lentiviral vectors, HEK293T cells were seeded at a density of 12 × 10^6^ cells in a 15 cm tissue culture plate pre-treated with Poly-L-Lysine solution (Sigma, St. Louis, MO). The cells were cultured in DMEM supplemented with 10% FBS, 1% penicillin/streptomycin, and 2 mM L-glutamine at 37°C in a 5% CO2 incubator. After 24 hours, the cells were transfected with engineered transfer plasmids containing the CAR sequence and shRNAs (**Figure 1A**) (10, 11) and two packaging plasmids, PMD2G and psPAX2, using the calcium phosphate precipitation method (13). The transfection medium was removed on next day, and fresh media was replaced. The viral particles were harvested 36 and 72 hours after transfection and centrifuged for 5 minutes at 500g to remove cell debris. The virus-containing media (VCM) was concentrated (100x) by ultracentrifugation at 25000 rpm for 2 hours at 4°C. The viruses were then resuspended in RPMI1640, aliquoted, and stored at −80°C. The concentrated viruses were titrated using the limiting dilution method on Jurkat cells. To determine the titer for each lentivirus, different volumes of concentrated VCM (0.01, 0.1, 1, 10, and 100 μL) were added to 0.5–1×10^6^/well Jurkat cells seeded in a 12-well cell culture plate. The expression of green fluorescent protein (GFP) was determined by flow cytometry 72 hours after transduction. The transduction unit per ml (TU/ml) was calculated using the following formula:

$$TU/mL=\frac{\%GFP\;positive\;cells\times number\;of\;cells}{Volume\;of\;virus\;in\;mL}$$

### Generation of CAR T cells

Healthy donor buffy coats were obtained from the Iranian Blood Transfusion Organization (IBTO) and used to isolate human peripheral blood mononuclear cells (PBMCs) through Ficoll-Paque gradient centrifugation (Sigma Aldrich, USA). T cells were then purified from the PBMCs using the negative selection method of magnetic-associated cell sorting (MACS) with the Human Pan T-Cell Isolation Kit II (Miltenyi Biotec). The purity of the isolated T cells was verified using FITC-conjugated anti-human CD3 (clone: UCHT1, #300406) by flow cytometry. T cells were activated using anti-CD3/CD28 antibody-coated beads (Gibco) at a 1:1 ratio and cultured in TexMACS medium supplemented with 10% human serum, 1% penicillin/streptomycin, and 100 IU/ml human recombinant IL2 (Miltenyi Biotec, Germany) at 37°C in 5% CO2. After 12–16 hours, to generate MSLN-CAR T cells and mock T cells, activated T cells were transduced with an appropriate volume of each concentrated lentiviral vector (at a multiplicity of infection (MOI) of 5) supplemented with 0.8 μL of polybrene (Santa Cruz Biotechnology, CA). The plates were spinfected at 2100 rpm at 32°C for 1 hour, followed by incubation at 37°C. Three hours later, 3 ml of fresh media was added to 1 ml in each well. T cells were debeaded 4–7 days after primary activation, and transduction efficiency was assessed using GFP-expressing T cells by flow cytometry.

### Evaluation of gene knockdown

Different MSLN-CAR T cells, including MSLN-CAR T cells, Tim3.KD.MSLN-CAR T cells, A2aR.KD.MSLN-CAR T cells and Tim3/A2aR.KD.MSLN-CAR T cells were generated. Percentage of A2aR and Tim3-positive Mock T cells, MSLN-CAR T cells, Tim3.KD.MSLN-CAR T cells, A2aR.KD.MSLN-CAR T cells and Tim3/A2aR.KD.MSLN-CAR T cells were determined before and 72 h after stimulation with HeLa cells by flow cytometry using anti-human adenosine A2aR Alexa Fluor® 647-conjugated antibody (R&D Systems) and anti-human Tim3 APC-conjugated antibody (BioLegend, USA-345,012) according to the manufacturer’s instructions. A2aR and Tim3 knockdown efficiency and the effects of knockdown of each receptor on the expression of other receptors were examined by flow cytometry.

### *In vitro* cytotoxicity assay

The HeLa cells were detached using 0.25% trypsin and 0.1% EDTA, followed by incubation at 37°C in 5% CO2. After incubation overnight, 1× 10^4^ HeLa cells were coincubated with different MSLN-CAR T cells and Mock T cells at target:effector ratios of 1:1, 1:5, 1:10, and 1:20 in 96-well U-bottomed plates with a final volume of 200 μl/well in complete TexMACS media. Assays were performed in the presence and absence of 1 μM adenosine analog (NECA, an adenosine receptor agonist) (Abcam). To distinguish between target and effector cells, MSLN-CAR T cells and mock T cells were labeled with CFSE dye. After 4 hours of coincubation, propidium iodide (PI) (1 μg/ml) was added as a viability dye to exclude dead cells just before flow cytometric analysis. Dead tumor cells were identified as CFSE-negative/PI-positive populations. To calculate the percentage of tumor cell lysis, the autolysis percentage of target cells was subtracted from the percentage of dead target cells that were incubated with effector cells.

### *In vitro* proliferation and cytokine production of CAR T cells

HeLa cells were detached and incubated overnight at 37°C in 5% CO2. Next day, HeLa cells were treated with 25 μg/ml mitomycin C (Sigma) for 30 min at 37°C, followed by washing. To evaluate proliferation, both mock T cells and all types of MSLN-CAR T cells were stained with 2.5 μM CFSE dye at room temperature for 8 min. After staining, the CFSE dye was quenched with FBS, and the cells were washed with complete RPMI medium. Next, 2×10^5^ CFSE-labeled effector cells were cocultured with equal ratios (1:1) of mitomycin-treated target cells in 48-well plates for 72 h in the presence and absence of NECA. All proliferation assays were conducted without exogenous IL-2. After 48 hours of coculture, the supernatant was collected and stored at -80°C for subsequent quantification of IFN-γ (KPG-HIFN-g), TNF-α (KPG-HTNF-a), and IL-2 (KPG-HIL-2​) using enzyme-linked immunosorbent assay (ELISA) according to the manufacturer’s instructions. After 72 h of coculture, the cells were harvested from the same wells and stained with anti-CD3-PerCP (BioLegend), and the percentage of CFSE dilution in CD3-positive gated cells was determined as the proliferation percentage.

### Flow cytometric analysis

All data were acquired with a BD FACSCalibur (BD Biosciences, San Jose, California) and analyzed using FlowJo (Treestar, v10.6.2) software. All assays were performed in duplicate and repeated at least two times.

### Animal experiments

Female athymic C57BL/6-nude mice aged 6–8 weeks were obtained from the Pasteur Institute of Iran (Amol Research Center) and were housed in individually ventilated cages (IVCs) in accordance with ARRIVE Guidelines 2.0. On day 0, 5×10^6^ HeLa cells (in 100 μL of PBS) were injected subcutaneously into the right flank of the nude mice. When the mean tumor volume reached 70 mm3, the tumor-bearing nude mice were divided into six groups of five mice each: PBS-1x group, Mock T-cell group, MSLN-CAR T-cell group, and Tim3.KD.MSLN-CAR T cell group, A2aR.KD.MSLN-CAR T cell group, and Tim3/A2aR.KD.MSLN-CAR T cell group. On the day 14 after tumor inoculation, each group was given an intratumoral injection of 1x10^7^ CAR T cells (14). The PBS-1x group and mock T cells group served as control groups and were respectively injected with 100 μL of PBS-1x and 1x10^7^ mock T cells. Tumor volumes were calculated every day or every other day using the formula length x width x height, and at the end, the mean tumor volumes in each group were measured and analyzed. The mice were euthanized when the tumor volume reached 2 cm^3^ (endpoint=2000 mm^3^).

### Immunohistochemistry (IHC) staining

At the endpoint, tumors were dissected, and an IHC assay for detection of mesothelin expression on the tumor tissue was conducted according to a previously published procedure (15). Briefly, antigen retrieval was performed with a microwave oven (850-watt power for 5 min, and then 340-watt power for 11 min) in Tris-EDTA buffer at pH 9. The anti-MSLN antibody (Abcam, ab96869) was applied at a final concentration of 0.5 micrograms per milliliter and incubated overnight at 4 degrees Celsius. Final visualization of the IHC staining were utilized with Mouse/Rabbit HRP detection reagent (Diagnostic Biosystems, cat. no.: MRUHRP100), followed by counterstaining with hematoxylin. An isotype control antibody (Biolegend, 910806) was used to check the specificity of the MSLN antibody staining.

### Statistics

All statistical analyses were performed with GraphPad Prism software. The sample size in the animal experiments was predetermined according to Hong’s study (16). Analysis of variance (ANOVA) followed by Tukey’s *post hoc* comparisons tests was used to reveal differences among all groups. Data are presented as the mean ± SD. p < 0.05 was considered statistically significant.

## Results

### Production and titration of lentiviral vectors

In this study, various lentiviral vectors containing either empty backbone, MSLN-CAR, Tim3-shRNA-MSLN-CAR, or A2aR-shRNA-MSLN-CAR sequences were used (**Figure 1A**). The HEK293T transfection method could efficiently produce lentiviral particles (**Figure 1B**). To evaluate the transduction efficacy, Jurkat cells were transduced with lentiviral particles, and the percentage of GFP expression was measured using flow cytometry (**Figure 1C**). The titration formula, as described in the methods section, was used to determine transduction units per milliliter, which was found to be in a range from 10^8^ to 10^9^ TU/ml. These findings confirmed the successful engineering and production of lentiviral particles for generation and exploring the therapeutic potential of MSLN-CAR, Tim3-shRNA-MSLN-CAR, and A2aR-shRNA-MSLN-CAR T cells *in vitro* and *in vivo*.

### MSLN-CAR T cells and KD.MSLN-CAR T cells were efficiently generated using lentiviral transduction

**Figure 2:**
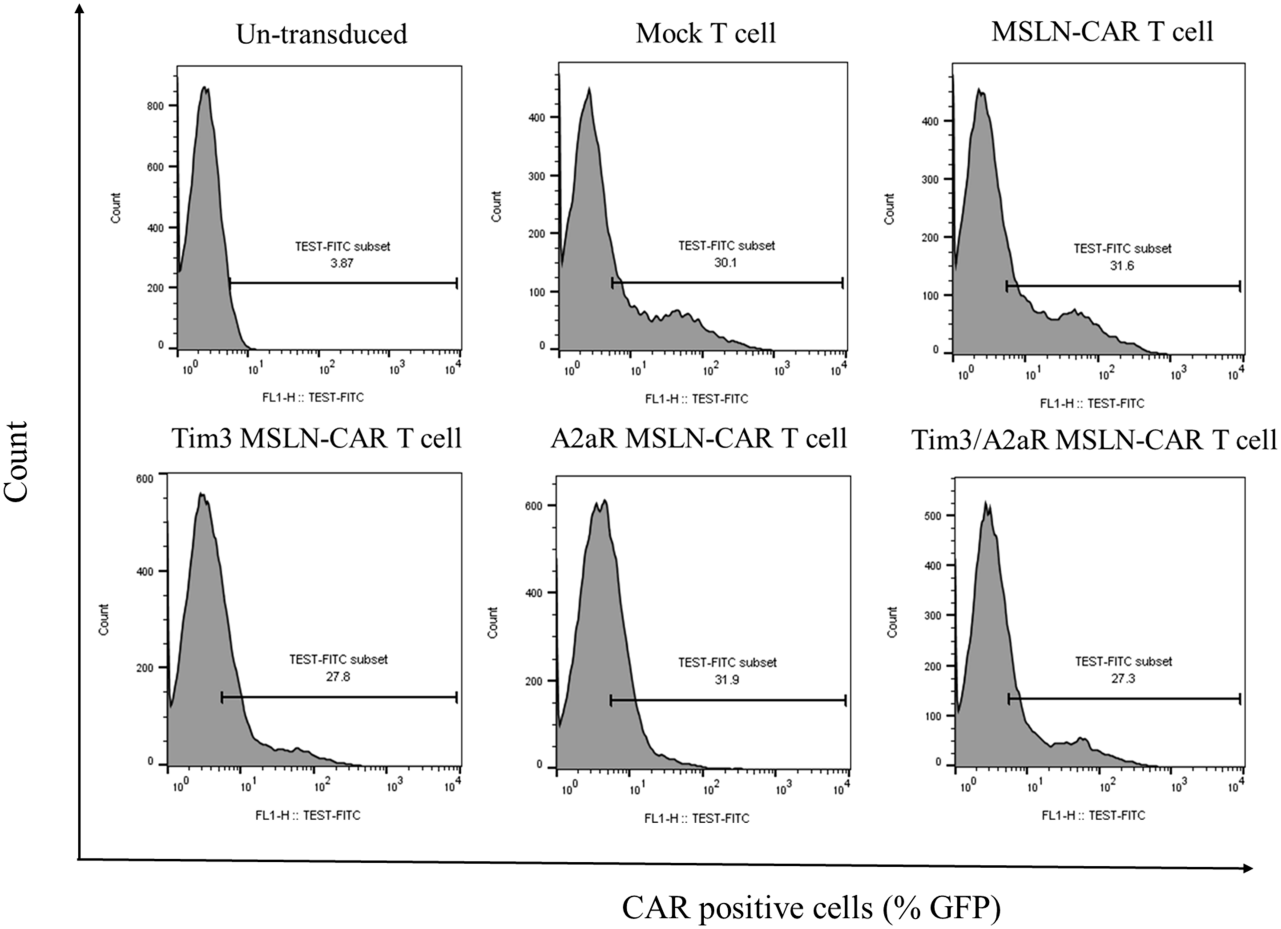
Generation of different types of MSLN-CAR T cells. Flow cytometry histogram plots show the percentage of CAR-positive T cells generated from one donor 4 days after transduction of anti-CD3/CD28-activated T cells with the indicated volume of lentiviral vectors at an MOI of ~5. Untransduced T cells refer to T cells that did not undergo transduction with lentiviral vectors. Mock T cells were transduced with a pCDH-CMV-MCS-EF1 vector without the MSLN-CAR sequence. MSLN-CAR T cells refer to T cells transduced with a lentivector containing the anti-mesothelin CAR. Tim3.KD.MSLN-CAR T cells refer to T cells containing both MSLN-CAR and Tim3 shRNA. A2aR.KD.MSLN-CAR T cells refer to T cells containing both MSLN-CAR and A2aR shRNA. Tim3/A2aR.KD.MSLN-CAR T cells were cotransduced with half of the indicated volume of lentivectors containing MSLN-CAR and Tim3 shRNA and MSLN-CAR and A2aR shRNA.

To investigate the effect of single and double knockdown of Tim3 and A2aR on the function of MSLN-CAR T cells, activated T cells were transduced (MOI of 5) with four types of second-generation MSLN-CAR lentiviral vectors, including MSLN-CAR, Tim3-shRNA-MSLN-CAR, A2aR-shRNA-MSLN-CAR, and Tim3/A2aR-shRNA-MSLN-CAR. Tim3/A2aR-KD-MSLN-CAR T cells were generated by simultaneously cotransduction of activated T cells with two types of lentiviral viruses containing Tim3-shRNA-MSLN-CAR and A2aR-shRNA-MSLN-CAR sequences. Mock T cells were used as a control group. Since all CAR and empty constructs contained GFP sequences, CAR transduction efficiency was determined by analyzing GFP expression using flow cytometry. Flow cytometry analysis indicated that 20–30% of the transduced T cells were CAR positive (**Figure 2**).

### Knockdown of Tim3 or A2a receptors had no reciprocal effect on their expression

Next, we investigated the expression of Tim3 and A2aR at the baseline level in peripheral blood T cells and in different types of MSLN-CAR T cells prior to and after coculture with mesothelin-positive tumor cells using flow cytometry. As shown in **Supplementary Figure 1A**, A2aR expression was increased on CAR T cells post stimulation, both with anti-CD3/28 antibodies and in coculture with mesothelin-positive HeLa cells. Our findings revealed that A2aR shRNA significantly reduces the overall expression of A2aR in CAR T cells that carried the anti-A2aR shRNA sequences, including A2aR.KD.MSLN-CAR T cells and Tim3/A2aR.KD.MSLN-CAR T cells relative to MSLN-CAR T cells prior to stimulation with tumor cells (**Supplementary Figure 1B**). However, in all CAR T-cell groups except for A2aR.KD.MSLN-CAR T cells, the percentage of A2aR-positive cells was increased significantly after stimulation with HeLa cells (**Supplementary Figure 1B**). Moreover, Tim3 knockdown had no effect on A2aR expression in Tim3.KD.MSLN-CAR T cells (**Supplementary Figure 1B**).

Although there was an increase in Tim3-positive CAR T cells after coculture with HeLa cells in all groups of CAR T cells, this increase was not significant (**Supplementary Figures 1C, D**). Furthermore, in Tim3.KD.MSLN-CAR T cells and Tim3/A2aR.KD.MSLN-CAR T cells, Tim3 shRNA could decrease the percentage of Tim3-expressing cells, but this effect was not significant (**Supplementary Figure 1D**). Additionally, genetic targeting of A2aR did not significantly affect the frequency of Tim3-positive cells (**Supplementary Figure 1D**). Overall, these findings demonstrate the efficient knockdown of Tim3 and/or A2aR in MSLN-CAR T cells and highlight an independent link between Tim3 and A2aR expression in CAR T cells.

### Simultaneous knockdown of Tim3 and A2a receptors enhanced MSLN-CAR T cell antitumor function

To assess the *in vitro* antitumor function of CAR T cells, the ability of CAR T cells to proliferate, kill, and produce cytokines in response to HeLa cells was measured. To investigate the effect of A2aR knockdown on CAR T cell function, we compared the functional capacity of CAR T cells in the absence and presence of NECA (an adenosine receptor agonist). Additionally, previous studies have shown that galectin-9, a Tim3 ligand, is expressed by HeLa cells (17–19). Prior to conducting functional experiments, using flow cytometry we assessed the expression of mesothelin as a tumor target antigen in HeLa and Panc-1 cell lines. Our data analysis showed that mesothelin is expressed in HeLa cells (approximately 60%), while Panc-1 cells do not express this target antigen (**Supplementary Figure 2A**).

**Figure 3:**
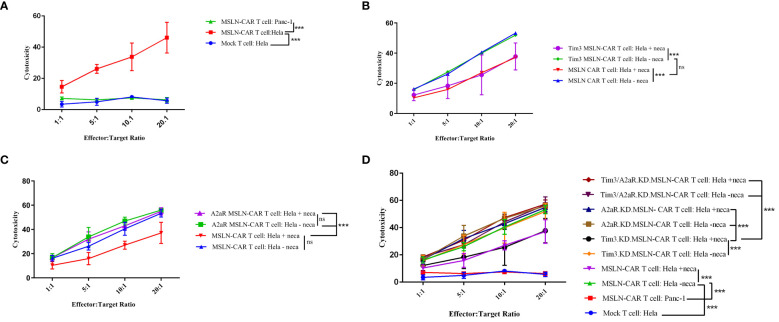
Antigen-dependent cytotoxicity of MSLN-CAR T cells. Line plots comparing the average percentage of dead HeLa target cells coincubated with MSLN-CAR T cells, Tim3.KD.MSLN-CAR T cells, A2aR.KD.MSLN-CAR T cells, Tim3/A2aR.KD.MSLN-CAR T cells and mock T cells at four different effector-to-target ratios (1:1, 5:1, 10:1, and 20:1) in the absence or presence of 1 μM NECA for 4 hours **(A-D)**. The cells were stained with propidium iodide (PI) dye. Data are representative of two independent experiments, each performed in duplicate. Two-way ANOVA followed by Tukey’s *post hoc* test was used for statistical analysis. P< 0.05 was considered statistically significant. (***P< 0.001). NECA: 5′-(N-ethylcarboxamido) adenosine; SD: standard deviation; MSLN-CAR T cells: fully human anti-mesothelin CAR T cells; Tim3.KD.MSLN-CAR T cells: MSLN-CAR T cells containing Tim3 shRNA; A2aR.KD.MSLN-CAR T cells: MSLN-CAR T cells containing A2aR shRNA; Tim3/A2aR.KD.MSLN-CAR T cells: MSLN-CAR T cells containing both Tim3 and A2aR shRNA; Mock T cells: T cells containing an empty vector.

First, we measured the effect of genetic targeting of A2aR and Tim3 on CAR T cells cytotoxic ability. We analyzed cytotoxicity using a gating strategy that considered the population of CFSE-negative/PI-positive cells as the percentage of lysed tumor cells (**Supplementary Figure 2B**). Our results demonstrated that MSLN-CAR T cells are capable of specifically recognizing and killing mesothelin-positive tumor cells (**Figure 3A**). Additionally, Tim3 knockdown had no significant effect on the cytotoxic potential of MSLN-CAR T cells (**Figure 3B**). In contrast, A2aR targeting in MSLN-CAR T cells could preserve CAR T cell cytotoxicity in the presence of NECA (**Figure 3C**), which was previously shown by Masoumi et al. (10). Altogether, A2aR and simultaneous A2aR/Tim3 knockdown in MSLN-CAR T cells were shown to enhance the killing potential of these cells in an immunosuppressive milieu (**Figure 3D**).

**Figure 4:**
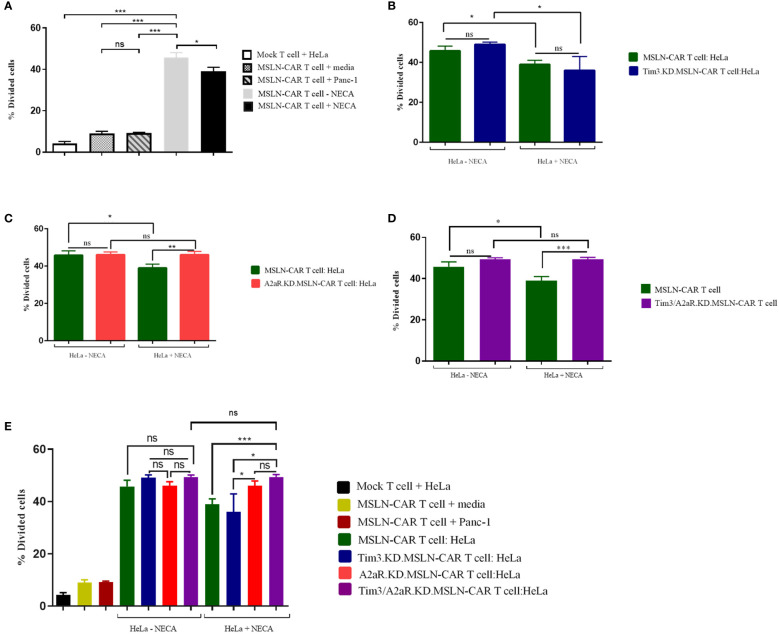
Proliferation potency of MSLN-CAR T cells in the presence of antigen and adenosine. 2 × 10^5^ CAR T cells from four groups, including MSLN-CAR T cells, Tim3.KD.MSLN-CAR T cells, A2aR.KD.MSLN-CAR T cells, and Tim3/A2aR.KD.MSLN-CAR T cells were labeled with CFSE dye and cocultured with mitomycin C-treated HeLa cells at a 1:1 ratio for 72 hours in the absence or presence of 1 μM NECA. Mock T cells containing an empty vector were used as control cells. The bar graphs show the percentage of divided cells between MSLN-CAR T cells (in presence and absence of Neca) and mock T cells against media and mesothelin positive (HeLa) and negative (Panc-1) cancer cells **(A)**. Antigen specific proliferation of MSLN-CAR T cells and Tim3.KD.MSLN-CAR T cells in presence and absence of Neca **(B)**. Antigen specific proliferation of MSLN-CAR T cells and A2aR.KD.MSLN-CAR T cells in presence and absence of Neca **(C)**. Antigen specific proliferation of MSLN-CAR T cells and Tim3/A2aR.KD.MSLN-CAR T cells in presence and absence of Neca **(D)**, and among all groups **(E)**. The data are presented as the mean ± standard deviation (SD). Mean comparisons were performed using Brown-Forsythe and Welch ANOVA followed by Dunnett T3’s *post hoc* test. Statistical significance was set at P< 0.05. The results represent two independent experiments (*P< 0.05, **P< 0.01, and ***P< 0.001). NECA, 5′-(N-ethylcarboxamido) adenosine; SD, standard deviation; MSLN-CAR T cells, fully human anti-mesothelin CAR T cells; Tim3.KD.MSLN-CARs, T cells containing MSLN-CAR and Tim3 shRNA; A2aR.KD.MSLN-CARs, T cells containing MSLN-CAR and A2aR shRNA; Tim3/A2aR.KD.MSLN-CARs, T cells containing MSLN-CAR and both Tim3 and A2aR shRNA; Mock T, T cells containing an empty vector.

Second, we aimed to assess the effect of A2aR and/or Tim3 knockdown on the ability of MSLN-CAR T cells to proliferate in response to mesothelin-positive tumor cells. The gating strategy related to the proliferation assay is shown in **Supplementary Figure 2C**. Similar to our cytotoxicity results, we observed that manufactured MSLN-CAR T cells are able to divide and proliferate in presence of mesothelin-positive cancer cells (**Figure 4A**) in an antigen-specific manner. Moreover, Tim3 knockdown showed no significant increase in CAR T cell proliferative capacity (**Figure 4B**). Nevertheless, targeting A2aR and A2aR/Tim3 was observed to be effective in preserving MSLN-CAR T cell proliferation in the presence of NECA (**Figures 4C–E**).

**Figure 5:**
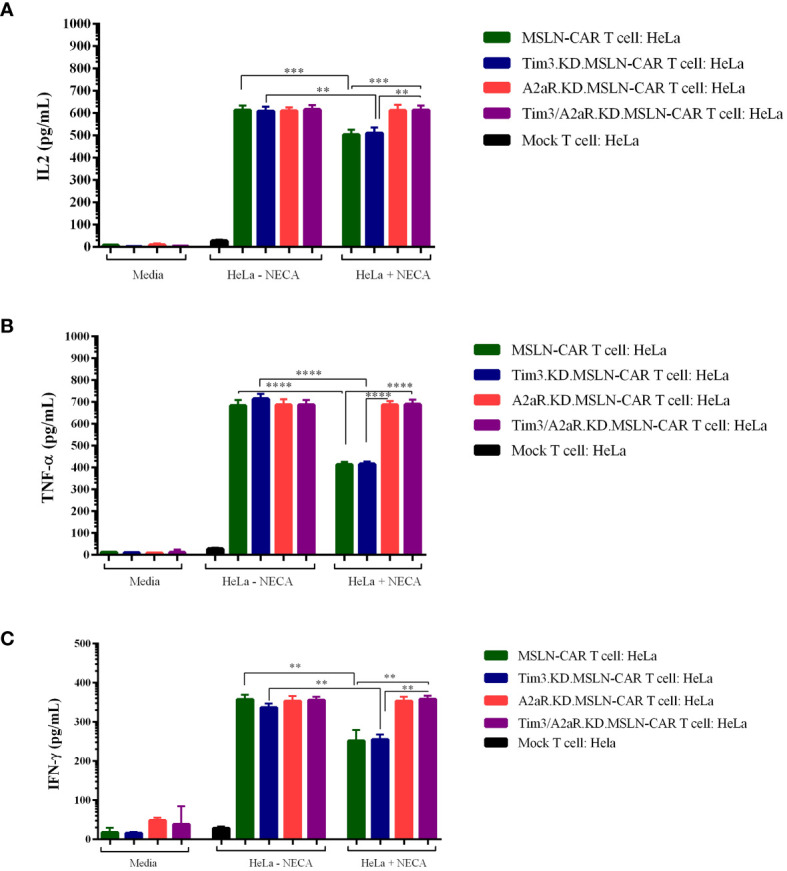
Cytokine production of different types of MSLN-CAR T cells. Fully human anti-mesothelin CAR T cells (MSLN-CAR T cells), Tim3 knockdown MSLN-CAR T cells (Tim3.KD.MSLN-CAR T cells), A2aR knockdown MSLN-CAR T cells (A2aR.KD.MSLN-CAR T cells), Tim3 and A2aR knockdown MSLN-CAR T cells (Tim3/A2aR.KD.MSLN-CAR T cells), and mock T cells containing empty vector were cocultured with HeLa target cells at a 1:1 ratio or media in the absence or presence of 1 μM NECA (5′-Nethylcarboxamido adenosine). After 48 hours, the supernatant was harvested, and the concentrations of IL2 **(A)**, TNFα **(B)**, and IFN-γ **(C)** cytokines were measured using ELISA. Data are presented as the mean ± standard deviation (SD) from two independent experiments. Mean comparisons were performed using one-way ANOVA followed by Tukey's *post hoc* test, with P< 0.05 considered statistically significant (**P< 0.01, ***P< 0.001, and ****P< 0.0001). MSLN-CAR T cells: fully human anti-mesothelin CAR T cells; Tim3.KD.MSLN-CARs: MSLN-CAR T cells containing Tim3 shRNA; A2aR.KD.MSLN-CARs: MSLN-CAR T cells containing A2aR shRNA; Tim3/A2aR.KD.MSLN-CARs: MSLN-CAR T cells containing both Tim3 and A2aR shRNA; Mock T cells: T cells containing empty vector.

Finally, we assessed the ability of gene knockdown MSLN-CAR T cells to produce cytokines (TNF-a, IL-2, and IFN-γ) in the presence and absence of NECA. Similar to previous studies (10, 11), we observed that NECA can suppress cytokine production from MSLN CAR T cells (**Figures 5A–C**). Interestingly, targeting A2aR and Tim3 was shown to significantly enhance cytokine production by MSLN-CAR T cells in the presence of NECA.

Altogether, although Tim3 shRNA reduced Tim3 expression, this reduction did not improve MSLN-CAR T cell function. The antitumor functions of Tim3.KD.MSLN-CAR T cells, as well as MSLN-CAR T cells, including cytotoxicity, proliferation, and cytokine production, significantly decreased in the presence of NECA. A2aR knockdown in A2aR.KD.MSLN-CAR T cells could maintain CAR T cell function in the presence of NECA, as previously reported by Masoumi et al. Since the main purpose of our *in vitro* study was to evaluate the effects of double knockdown of Tim3 and A2a receptors on the antitumor function of MSLN-CAR T cells, we compared the antitumor capacity of Tim3/A2aR.KD.MSLN-CAR T cells with a single KD.MSLN-CAR T cells. We found that double KD.MSLN-CAR T cells showed similar antitumor functions in the presence and absence of NECA. Additionally, although double knockdown of Tim3 and A2a receptors significantly increased the cytotoxicity, proliferation, and cytokine production capacity of Tim3/A2aR.KD.MSLN-CAR T cells relative to Tim3.KD.MSLN-CAR T cells and MSLN-CAR T cells in the presence of NECA, there was no significant difference between double KD.CAR T cells and A2aR KD.CAR T cells (**Figures 3**–**5**). In summary, these findings highlight the potential of A2aR knockdown to improve the antitumor function of MSLN-CAR T cells, especially in the presence of NECA, and the lack of a significant synergistic effect of double knockdown of Tim3 and A2a receptors on the antitumor function of MSLN-CAR T cells.

### MSLN-CAR T cells and Tim3/A2aR.KD.MSLN-CAR T cells showed a significant decrease in tumor volume *in vivo*

**Figure 6:**
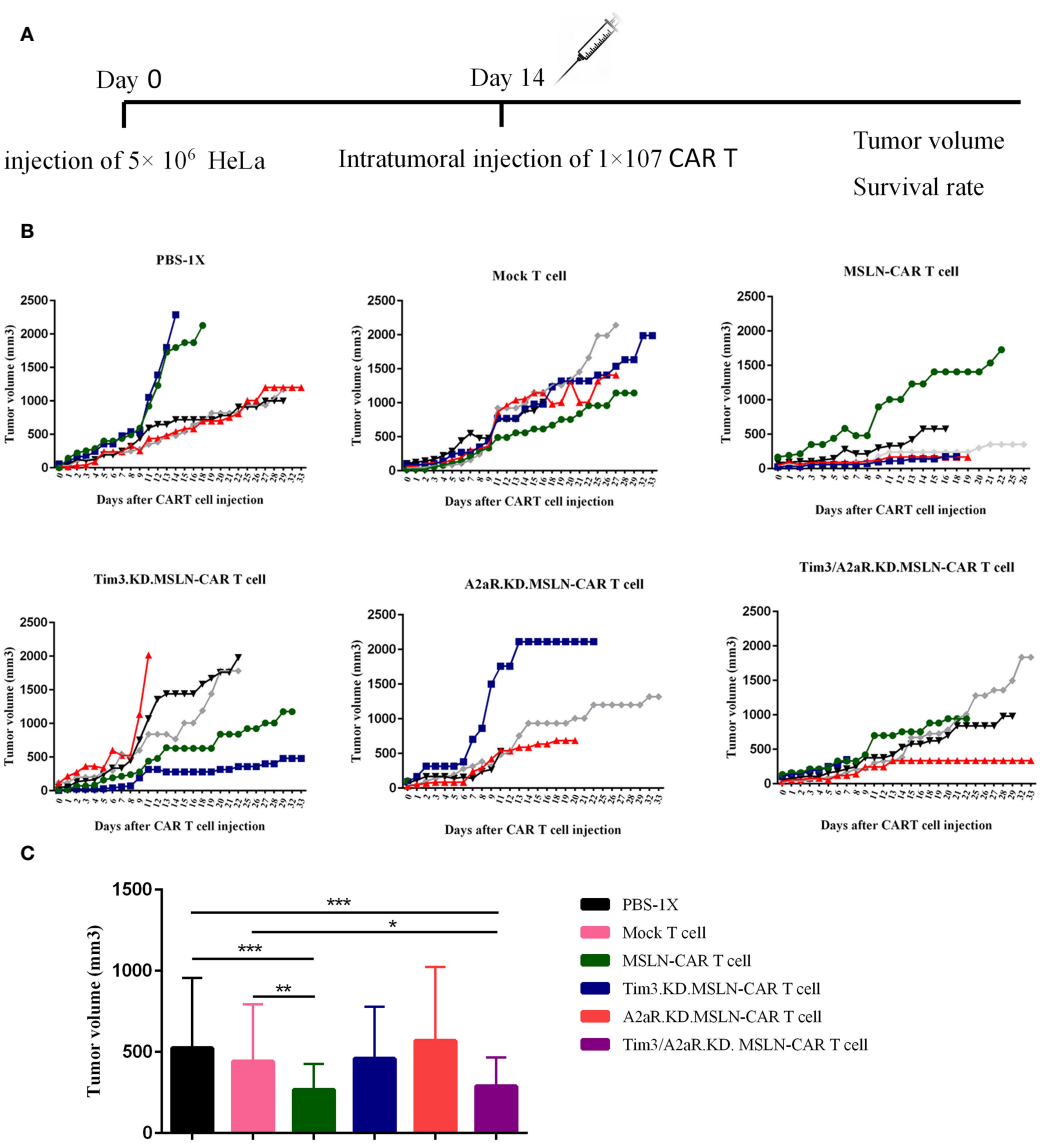
The effect of CAR T cells on tumor growth. The effect of CAR T cells on tumor growth was evaluated in six groups of C57BL/6-nude mice (n=5) treated with 1x10^7^ different CAR T cells, including MSLN-CAR T cells, Tim3.KD.MSLN-CAR T cells, A2aR.KD.MSLN-CAR T cells, and Tim3/A2aR.KD.MSLN-CAR T cells. PBS-1X and Mock T cells were used as control groups. Tumor volume was measured on day 0 (the time of intervention) and every 24–48 hours **(A)**. Line plots depict the rate of tumor growth over time for each of the five mice in each group **(B)**. Each color represents the tumor growth trend curve for one mouse. The bar graph displays the tumor volume for each group **(C)**. Data are presented as the mean ± SD. Mean comparisons were performed using two-way ANOVA followed by Tukey's *post hoc* test. A P value less than 0.05 was considered statistically significant (*P< 0.05, **P< 0.01, ***P< 0.001). The results are representative of two independent experiments. The CAR T cells used in the study were fully human anti-mesothelin CAR T cells (MSLN-CAR T cells), T cells containing MSLN-CAR and Tim3 shRNA (Tim3.KD.MSLN-CAR T cells), T cells containing MSLN-CAR and A2aR shRNA (A2aR.KD.MSLN-CAR T cells), T cells containing MSLN-CAR and both Tim3 and A2aR shRNA (Tim3/A2aR.KD.MSLN-CAR T cells), and mock T cells containing an empty vector. The PBS-1x group received phosphate-buffered saline.

To determine the *in vivo* capacity of manufactured CAR T cells, we injected 5×106 HeLa cells into the right flank of nude mice, followed by intratumoral injection of different groups of gene knocked down MSLN-CAR T cells (**Figure 6A**). The expression of mesothelin in tumors was investigated using the IHC method. Tissues were resected and stained with rabbit IgG anti-human mesothelin. The obtained images showed that approximately 30–40% of tumor cells had mesothelin expression (**Supplementary Figure 3A**). To confirm the specificity, a rabbit IgG isotype control was used, which showed no positive reaction on the tumor cells (**Supplementary Figure 3B**). A human tumor of the fallopian tube with mesothelin expression was used as a positive control (**Supplementary Figure 3C**). Mouse liver tissue was used as a negative control for human mesothelin antigen (**Supplementary Figure 3D**).

**Figure 7:**
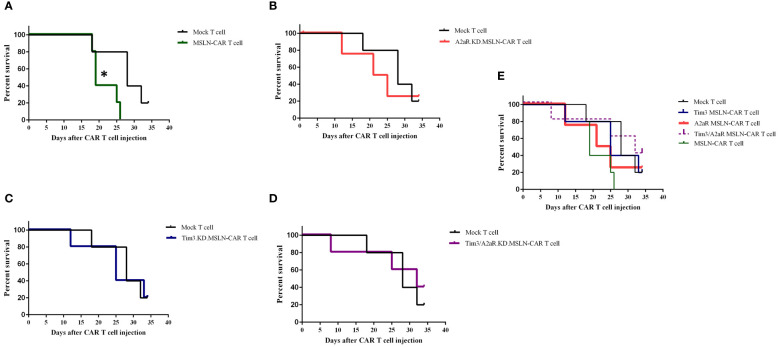
Impact of CAR T cell therapy on survival rates in C57BL/6-nude mice. Six groups of mice (n = 5) were treated with different CAR T cell therapies, including MSLN-CAR T cells and Tim3.KD.MSLN-CAR T cells, A2aR.KD.MSLN-CAR T cells, and Tim3/A2aR.KD.MSLN-CAR T cells, as well as control groups treated with PBS-1X and mock T cells. Kaplan-Meier plots show the comparison of percent survival between the mock T cell group and MSLN-CAR T cells **(A)**, A2aR.KD.MSLN-CAR T cells **(B)**, Tim3.KD.MSLN-CAR T cells **(C)**, Tim3/A2aR.KD.MSLN-CAR T cells **(D)**, and all groups combined **(E)**. The number of days the mice survived the intervention (day 0) was recorded, and the endpoint was defined as a tumor volume of 2000 mm^3^. The survival data were analyzed using a log-rank test. In the Kaplan-Meier plot for the MSLN-CAR T group, the symbol * indicates that mouse deaths occurred when tumor sizes ranged from 100–300 mm^3^.

To evaluate the antitumor capacity of the four groups of MSLN-CAR T cells compared to the mock T cell and PBS-1x control groups, tumor volume and survival rate were analyzed after intratumoral injection of CAR T cells. **Figure 6B** shows the changes in tumor volume over time in five mice in each treatment group. The results indicated that the mice were treated with a single KD.MSLN-CAR T cells (either Tim3 or A2aR KD) had a similar rate of tumor volume compared to the Mock T cell and PBS-1x control groups. Interestingly, MSLN-CAR T cells and Tim3/A2aR.KD.MSLN-CAR T cells showed a significant decrease in tumor volume compared to the controls (**Figure 6C**). Nevertheless, none of the treatment groups had a significant effect on the survival rate of the animal xenograft models (**Figures 7A–E**), the Tim3/A2aR KD.MSLN-CAR T cells partially improved the survival rate compared to the Mock T cells (**Figure 7B**). Although tumor progression was slower in the group of mice treated with MSLN-CAR T cells, a high percentage of mice with tumor sizes between 100–300 mm^3^ unexpectedly died in this group (**Figure 7A**). To gain a better understanding of these unexpected outcomes, further studies are needed.

## Discussion

Compared to hematological malignancies, the efficacy of CAR T cells as living drugs for treating solid tumors is more tricky and harder owing to various reasons. One prominent reason is an increased expression of inhibitory receptors, including Tim3, LAG3, PD-1, and A2aR, on the surface of CAR T cells after their infiltration into the immunosuppressive TME followed by excessive antigen stimulation (20). Tim3 and A2aR are considered as exhaustion markers of T cells, and their expression on CAR T cells reduces the ability of these cells to kill tumor cells, proliferate, and produce cytokines (4, 21). Several studies have shown that suppressing these inhibitory receptors using strategies such as shRNA-mediated knockdown, CRISPR-Cas9-mediated knockout, or blocking their interaction with their cognate ligands can restore the antitumor function of CAR T cells (22).

In previous studies, Masoumi et al. (10) and Jafarzadeh et al. (11) manufactured two MSLN-CAR T cells in which Tim3 or A2aR receptors were knocked down using shRNA. The Tim3 molecule, also known as HAVCR2 (Hepatitis A Virus Cellular Receptor 2), belongs to the Tim family located on human chromosome 5 (23). This molecule is a transmembrane protein type I that contains an extracellular domain, a transmembrane region, and a cytoplasmic tail in the C-terminus (24). When Tim3 binds to its ligands, such as galectin-9, it leads to phosphorylation of tyrosine motif (Y256 and Y263) through IKT and src kinases and releases the BAT3 chaperone molecule from the cytoplasmic tail of Tim3. This ultimately inhibits the PI3K/AKT and LCK/ZAP70 signaling pathways in T cells (25). The adenosine molecule, which increases under the influence of hypoxia in the TME, has four receptors, including A1R, A2aR, A2bR, and A3R. Among them, the A2aR receptor binds to adenosine with higher affinity and suppresses the function of lymphocytes (5). The A2aR receptor is a member of the G protein-coupled receptors that, following binding to adenosine, activates adenylate cyclase through the stimulatory subunit (Gas/olf) and increases the intracellular level of cAMP. The increase in cAMP levels through activation of protein kinase A and phosphorylation of the CREB transcription factor leads to inhibition of signaling pathways related to lymphocyte function (26). Therefore, although Tim3 and A2aR both inhibit T lymphocyte function, they exert their functions through distinct signaling pathways.

One of the primary objectives of this study was to explore the impact of simultaneous genetic targeting of Tim3 and A2a receptors on the function of MSLN-CAR T cells. Previous research has demonstrated that concurrent inhibition of two inhibitory receptors can enhance the antitumor function of T cells or CAR T cells (27–30). Therefore, we hypothesized that Tim3/A2aR double knockdown MSLN-CAR T cells would exhibit improved antitumor function compared to single knockdown cells. In this study, in addition to synthesizing Tim3 or A2aR single knockdown MSLN-CAR T cells, we generated Tim3/A2aR double knockdown MSLN-CAR T cells. We compared antitumor functions of double knockdown MSLN-CAR T cells with single knockdown and unmodified MSLN-CAR T cells.

Our study demonstrated that NECA, an analog of adenosine, inhibits the function of MSLN-CAR T cells that lack A2aR shRNA sequences, such as MSLN-CAR T and Tim3.KD.MSLN-CAR T cells. However, A2aR (A2aR.KD.MSLN-CAR T cells)and Tim3 (Tim3/A2aR.KD.MSLN-CAR T cells) preserved their antitumor functions in the presence of adenosine. This finding supports previous research demonstrating that adenosine inhibits the antitumor function of T cells through A2a receptors and that inhibition of A2a receptors can reverse adenosine-induced suppression of CAR T cells (10, 30, 31). However, while Tim3/A2aR double knockdown MSLN-CAR T cells significantly improved antitumor function *in vitro*, our expectation was not met, as their antitumor function was not significantly different from that of A2aR single knockdown CAR T cells. Additionally, Tim3 targeting was not able to improve the function of Tim3 knockdown MSLN-CAR T cells compared to MSLN-CAR T cells, even though Tim3 shRNA reduced Tim3 expression. Other studies have shown that inhibiting one receptor can increase the expression of other inhibitory receptors in a compensatory manner (29, 32), which may be the case here. Therefore, to better understand this finding, we investigated the interplay between A2aR and Tim3 expression on the surface of CAR T cells. We found that the expression of A2aR was significantly reduced in CAR T cells with A2aR shRNA3 compared to MSLN-CAR T cells without A2aR shRNA, consistent with previous findings (10). However, the Tim3-shRNA failed to effectively and significantly downregulate Tim3 molecule, which is opposed to a previous study (11). This inconsistency may be due to the unique characteristics of T lymphocytes in different donors. Activation of CAR T cells has been shown to increase the expression of inhibitory receptors such as Tim3 (11, 28, 33) and A2aR (10, 30, 34) in previous studies, and our study also showed that the expression of Tim3 and A2aR molecules both increased on the surface of MSLN-CAR T cells after stimulation with HeLa cells. Interestingly, knockdown of Tim3 or A2aR did not affect their reciprocal expression, whereas previous studies have shown that inhibiting one inhibitory receptor can increase the compensatory expression of other inhibitory receptors. For instance, Leone et al. showed that A2aR inhibition with CPI-444 downregulates the expression of inhibitory receptors, including Tim3, Lag3, and PD1 (35). In another study, it was observed that PD1 inhibition increases the compensatory expression of Tim3 (36). Nevertheless, these differences may be due to differences in the source of T cells, the method of inhibition (genetic vs. pharmacological inhibition), and testing context (*in vitro* versus *in vivo)*. Alternatively, Tim3 knockdown may upregulate inhibitory receptors other than A2aR and neutralize the effect of Tim3 downregulation on improving the function of Tim3 knockdown cells, including Tim3.KD.MSLN-CAR T and Tim3/A2aR.KD.MSLN-CAR T cells. Therefore, further studies are required to profile expression of other immune inhibitory receptors upon Tim3 or A2aR downregulation.

Although MSLN-CAR T cells have shown considerable antitumor potential *in vitro* and *in vivo*, we observed that mice with very small tumor volumes (100–300) died between 17 and 19 days in our *in vivo* experiments. The cause of their death was not due to increased tumor volume but rather may be attributed to the occurrence of lymphoproliferative disorder caused by the antitumor function and heightened proliferation of MSLN-CAR T cells (37). However, further studies are needed to gain understanding of this outcome. These studies should focus on investigating the potential toxic effects of CAR T cell therapy, as well as the dynamic interplay between CAR T cells and the TME, including the occurrence of micrometastasis following CAR T cell therapy. Additionally, evaluating the immune response in mice treated with MSLN-CAR T cells, including the infiltration of immune cells into the tumor site and the production of inflammatory cytokines, would be beneficial.

In our *in vivo* experiments, Tim3 or A2aR single KD.MSLN-CAR T cells failed to decrease tumor volume or improve the survival rate in nude mice. This lack of efficacy for Tim3 KD.MSLN-CAR T cells partially aligns with their *in vitro* data, where they didn’t display substantial antitumor activity. A2aR knockdown, however, showed promise *in vitro* by enhancing CAR T cell function. However, this effect wasn’t observed *in vivo*. The complex TME likely contributes to this discrepancy (38). TME comprises various immunosuppressive cells (i.e., Tregs, TAMs, and MDCSs) and molecules (i.e., IDO, PD-L1, and Prostaglandin E2) which are not present *in vitro* settings; these suppressive entities are able to hinder the observed *in vitro* antitumor function of CAR T cells in preclinical and clinical settings (39). One example of TME complexity is the presence of ectoenzymes CD39 and CD73. These enzymes, in addition to tumor cells, are highly expressed by Tregs, which convert ATP to adenosine. Adenosine binds to A2aR, inhibiting T cell function (40). M2 macrophages and neutrophils also express and utilize these enzymes (41, 42). Another potential explanation for the *in vivo* failure of A2aR KD.MSLN-CAR T cells relates to compensatory upregulation of other inhibitory receptors (29, 32). Blocking a single inhibitory receptor (like A2aR) might lead to increased expression of ligands for other inhibitory receptors like PD-1 and TIGIT (43, 44).

Although Tim3/A2aR double KD.MSLN-CAR T cells showed a significantly lower mean tumor volume than A2aR single KD.MSLN-CAR T cells, *in vitro* assays showed that Tim3/A2aR KD.MSLN-CAR T cells had a similar antitumor function as A2aR single KD.MSLN-CAR T cells. The inconsistency of the *in vitro* and *in vivo* findings can be attributed to the different amounts of lentivirus used to manufacture different types of CAR T cells. Since the A2a receptor has been shown to have dual effects on T lymphocyte function – inhibiting it on one hand and promoting survival signals on the other (45) – complete inhibition of this receptor may reduce the *in vivo* persistence of T or CAR T cells (46, 47). In our *in vivo* study, the discrepancy in the effectiveness of A2aR single KD.MSLN-CAR T cells and Tim3/A2aR double KD.MSLN-CAR T cells can be explained by the difference in the intensity of A2aR inhibition between the two types of CAR T cells. Future studies aiming to optimize intensity of A2aR knockdown on CAR T cells would be beneficial in understanding the dual role of A2aR signaling on CAR T cells antitumor function *in vitro* and *in vivo*.

## Conclusions

In conclusion, our study highlights the inhibitory role of Tim3 and A2a receptors on the function of MSLN-CAR T cells. Although Tim3/A2aR double knockdown MSLN-CAR T cells exhibited improved antitumor performance *in vitro*, their function was not significantly different from that of A2aR single knockdown CAR T cells. This suggests that compensatory mechanisms or other inhibitory receptors may counteract the effect of Tim3 knockdown. Further investigation is needed to elucidate the interplay between different inhibitory receptors and to optimize CAR T cell therapy for solid tumors. Additionally, understanding the potential toxic effects and the dynamic interaction between CAR T cells and the TME *in vivo* will be crucial for the development of effective and safe CAR T cell therapies in the future.

## Data availability statement

The original contributions presented in the study are included in the article/**Supplementary Material**. Further inquiries can be directed to the corresponding author.

## Ethics statement

The studies involving humans were approved by Research Ethics Committees of the School of Medicine, Tehran University of Medical Sciences [IR.TUMS.MEDICINE.REC.1399.857]. The studies were conducted in accordance with the local legislation and institutional requirements. The participants provided their written informed consent to participate in this study. The animal study was approved by Research Ethics Committees of the School of Medicine, Tehran University of Medical Sciences [IR.TUMS.MEDICINE.REC.1399.857]. The study was conducted in accordance with the local legislation and institutional requirements.

## Author contributions

TS: Data curation, Formal analysis, Investigation, Visualization, Writing – original draft. BA: Data curation, Formal Analysis, Investigation, Writing – original draft. ZS: Writing – original draft. HM: Conceptualization, Writing – review & editing. JH: Conceptualization, Funding acquisition, Supervision, Writing – review & editing.

## Supplementary material

Supplementary Figure 1 Evaluation of Tim3 and A2aR expression in different types of MSLN-CAR T cells. **(A, B)** Representative flow cytometry histograms show the percentage of A2aR **(A)**- and Tim3 **(B)**-positive cells in a population of peripheral blood T cells, mock T cells, MSLN-CAR T cells, and Tim3.KD cells. MSLN-CAR T cells, A2aR.KD. MSLN-CAR T cells, and Tim3/A2aR.KD. MSLN-CAR T cells from one donor before and after stimulation with the indicated stimulators. **(C, D)** Bar graphs represent the percentage of A2aR **(C)**- and Tim3 **(D)**-positive cells in mock T cells and four different CAR T cells, including MSLN-CAR T cells and Tim3.KD.MSLN-CAR T cells, A2aR.KD.MSLN-CAR T cells, and Tim3/A2aR.KD.MSLN-CAR T cells from two healthy donors. Intragroup comparison of the percentage of A2aR- or Tim3-positive cells in each group was analyzed before and after HeLa cell stimulation. Intergroup comparison of the percentage of A2aR- or Tim3-positive cells among different groups was also performed. The data are presented as the mean ± SD. Mean comparisons were performed using one-way ANOVA followed by Tukey's *post hoc* test, with P< 0.05 considered statistically significant. The results are representative of two independent experiments. (*P< 0.05, **P< 0.01, ***P< 0.001, and ****P< 0.0001). MSLN-CAR T cells: fully human anti-mesothelin CAR T cells, Tim3.KD.MSLN-CARs: T cells containing MSLN-CAR and Tim3 shRNA, A2aR.KD.MSLN-CARs: T cells containing MSLN-CAR and A2aR shRNA, Tim3/A2aR.KD.MSLN-CARs: T cells containing MSLN-CAR, Tim3 shRNA, and A2aR shRNA, Mock T: T cells containing empty vector.

Supplementary Figure 2 Gating Strategy for Assessing Cytotoxicity and Proliferation. **(A)** Histogram plots show the percentage of mesothelin-expressing cells in Panc-1 and HeLa cells from left to right. **(B)** The gating strategy for detecting dead target cells as a percentage of cytotoxicity involved labeling effector cells with carboxyfluorescein succinimidyl ester (CFSE) stain, followed by coculture with target cells for 4 h. Cells were then stained with propidium iodide (PI), and the population of CFSE-/PI+ cells was identified as dead target cells. **(C)** To determine the percentage of proliferation, after coculture of effector and target cells for 72 h, the mixed population was stained with PerCP-anti-CD3 antibody, and the percentage of CFSE dilution in the gated CD3-positive cells was calculated. This percentage corresponds to the percentage of proliferation.

Supplementary Figure 3 Mesothelin expression in cell line-derived tumors. Immunohistochemistry (IHC) was used to investigate mesothelin expression in cell line-derived tumor tissue. Images **(A, C)** demonstrate mesothelin expression in experimental tumors and fallopian tube human tumors, respectively. Image **(B)** displays IHC of experimental tumors with rabbit IgG as an isotype control, and image **(D)** shows IHC of mesothelin in mouse liver tissue. The brown color in Image A and C shows mesothelin expression.
